# Legacy perfluoro-alkyl substances impair LDL-cholesterol uptake independently from PCSK9-function

**DOI:** 10.1016/j.toxrep.2023.09.016

**Published:** 2023-09-22

**Authors:** Iva Sabovic, Maria Giovanna Lupo, Ilaria Rossi, Federica Pedrucci, Andrea Di Nisio, Stefano Dall’Acqua, Nicola Ferri, Alberto Ferlin, Carlo Foresta, Luca De Toni

**Affiliations:** a Unit of Andrology and Reproductive Medicine, Department of Medicine, University of Padova, Padova, Italy; b Department of Medicine, University of Padova, Padova, Italy; c Department of Pharmacological Sciences, University of Padova, Padova, Italy; d Veneto Institute of Molecular Medicine, Padova, Italy

**Keywords:** Environmental exposure, Endocytosis, Sphingosine, LDL-receptor, Triglycerides

## Abstract

Perfluoro-alkyl substances (PFAS) are pollutants, whose exposure was associated with altered levels of low-density lipoproteins (LDL) in humans. Here we investigated this clinical outcome in two groups of young male adults residing in areas of respectively low and high environmental exposure to perfluoro-octanoic-acid (PFOA). From the Regional Authority data on pollution areas, 38 not-exposed and 59 exposed age-matched participants were evaluated for serum levels of total cholesterol (Total-Chol), LDL-Chol, high-density lipoprotein cholesterol (HDL-Chol), triglycerides (Tgl) and chromatography quantified PFOA. Human hepato-carcinoma cell line HepG2 was exposed to PFOA or perfluoro-octane-sulfonate (PFOS), as legacy PFAAs, and C6O4 as new generation compound. Fluorimetry was used to evaluate the cell-uptake of labelled-LDL. Proprotein Convertase Subtilisin/Kexin 9 (PCSK9)-mediated LDL-receptor (LDL-R) degradation and sub-cellular localization of LDL-R were evaluated by western blot analysis. Serum levels of PFOA, were positively and significantly correlated with Total-Chol (*ρ* = 0.312, P = 0.002), LDL-Chol (*ρ* = 0.333, P = 0.001) and Tgl (*ρ* = 0.375, P < 0.001). Participants with high serum LDL-Chol and Tgl levels, according to the cardiovascular risk, were more prevalent in exposed compared to not-exposed subjects (respectively: 23.7% *vs* 5.3%, P = 0.023 and 18,6% *vs* 0%, P = 0.006). Exposure of HepG2 cells to PFOA or C6O4 100 ng/mL was associated with a significantly lower LDL uptake than controls but no major impact of any PFAAs on PCSK9-mediated LDL-R degradation was observed. Compared to controls, exposure to PFAS showed an unbalanced LDL-R partition between membrane and cytoplasm. Endocytosis inducer sphingosine restored LDL-R partition only in samples exposed to C6O4. These data suggest a novel endocytosis-based mechanism of altered lipid trafficking associated with the exposure to legacy PFAS.

## Highlights

• Exposure to legacy PFAS is associated with increased LDL levels.
• In vitro exposure to PFAS reduces LDL-uptake with no involvement of PCSK9.
• PFAS, and particularly legacy ones, stably alter cell trafficking of LDL-receptor.
• Endocytosis inducer restores LDL-uptake only for new generation-PFAS representative.

## Graphical Abstract

**Figure:**
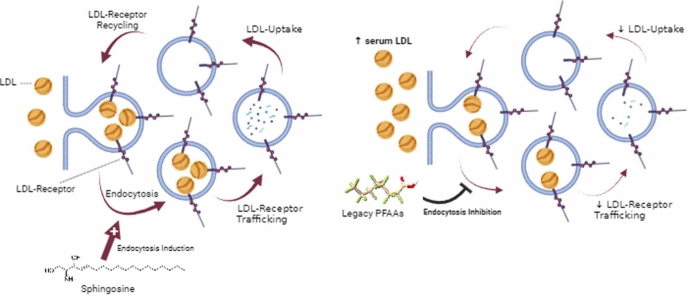

## 1 Introduction

Perfluoro-alkyl substances (PFAS) are a group of molecules widely used in a variety of industrial applications, from food packaging, textiles, water-repellent paper to household cleaners. Their chemical characteristics, featured by the high degree of fluoro-hydrogen substitution, make them poorly biodegradable and, therefore, highly persistent in the environment and in animal tissues [1]. This has led to the detection of considerable concentrations in wildlife and populations around the world [2]. It has been observed that serum half-life of PFAS in rodent models is in the order of 20–30 days. In contrast, available data in human show an estimated serum half-life ranging from 2.6 to 8.5 years [3], [4]. The environmental exposure to these widespread pollutants, particularly to perfluoro-octanoic acid (PFOA) and perfluoro-octane-sulfonate (PFOS), has been associated with several human health consequences, including metabolic derangements and increased cardiovascular mortality [5].

Increased serum levels of low-density lipoprotein-cholesterol (LDL-Chol) is unanimously recognized as a cardiovascular risk factor [6]. Several studies have demonstrated a thorough association between serum levels of LDL-Chol and the development and progression of atherosclerosis and cardiovascular mortality, mainly related to the deposit of oxidized LDL on the arteries walls [5], [6]. Hypercholesterolemia can be the result of non-genetic and genetic factors. In the former case, long term exposure to lifestyle factors, such as a high fat diet, poor physical activity, or environmental factors, such as the occupational exposure to environmental pollutants, associates with hypercholesterolemia and the consequent cardiovascular risk [7]. On the other hand, familial hypercholesterolemia (FH) is a genetic disorder associated with dominant mutations in the LDL receptor (LDL-R) gene and/or in key proteins involved in LDL-R endocytic recycling and resulting in increased serum levels of LDL-Chol [8], [9]. In this regard, Proprotein Convertase Subtilisin/Kexin 9 (PCSK9) is a recognized molecule able to drive the balance of LDL-R trafficking towards its lysosomal degradation [10]. Interestingly, up to 0.1–2% of subjects with clinical diagnosis of FH bear pathogenic variants in the PCSK9 gene, highlighting its role on LDL-R trafficking and in cholesterol metabolism [11].

Prolonged exposure to air pollutants or other environmental chemicals, such as PFAS, are associated with altered lipid profile and energy metabolism in both animal and human models, increasing the risk of cardiovascular issues in the exposed population [12], [13], [14], [15]. PFOA and PFOS have been suggested to exert hyperlipidemic effects through the activation of PPARα in animal models, however other mechanisms have been suggested, including the alteration of oxidative stress-related pathways and the modification of the DNA methylation pattern [16], [17], [18], [19].

In this study, we evaluated the serum lipid pattern, in relation to serum PFOA levels, in two groups of subjects residing in two areas at, respectively, high and low environmental exposure to PFAS. Furthermore, investigated the possible involvement of PCSK9 in the PFAS-associated derangement of LDL-R trafficking in a human hepato-carcinoma HepG2 cell line. In addition, we investigated the possible differential pattern associated with the exposure to legacy PFAS, such as PFOA and PFOS, and new generation compounds, such as C_6_O_4_ which has been recently purposed as PFOA substitute [20].

## 2 Materials and methods

### 2.1 Chemicals

Native compounds perfluoro-octanoic acid (PFOA), perfluoro-octan-sulphonic acid (PFOS) and the acetic acid, 2,2-difluoro-2-((2,2,4,5-tetrafluoro-5(trifluoromethoxy)− 1,3-dioxolan-4-yl)oxy)-ammonium salt (1:1) (C6O4) were all purchased from Wellington Laboratories (Southgate, Ontario, Canada). Stock standard solutions, all at the concentration of 50 mg/mL, were prepared in methanol and stored at the 4 °C until use. Bodipy-dye-labelled human low-density lipoproteins (cat# L3483) and Subcellular protein fractionation kit (cat# 78840) were purchased form Thermo Fisher Scientific (Life Technologies Italia, Monza, Italy). Human recombinant PCSK9 (cat# 20631) was purchased from Cayman Chemical (Ann Arbor, Michigan, USA). Anti LDL-Receptor rabbit polyclonal (Clone: EP1553Y; cat# ab52818) was purchased from Abcam (Cambridge, UK). Sphingosine (cat# 860490 P) and protease inhibitor cocktail powder (cat#P2714) were purchased from Merk Life Science S.r.l. (Milano, Italy).

### 2.2 Subjects

The study was performed as part of the continuous survey program for the reproductive health in the high schools of Padova and surroundings (Veneto region, northeast of Italy), aimed to address the early diagnosis of possible risk factors and diseases of the male reproductive system. During the dissemination interventions, adult students in the most advanced classes were offered spontaneous participation in the study. In general, more than 95% of the students joined the initiative. In this framework, 97 subjects aged 18–24 voluntarily agreed to complete the cross-sectional study between October 2018 and June 2019. Written informed consent was obtained from all subjects, and the study was approved by the Research Ethics Committee of the University Hospital of Padua (N. 2208 P). Participants did not receive any reimbursement. The investigation was performed according to the principles of the Declaration of Helsinki. Exclusion criteria included: acute or chronic established cardiovascular diseases, such as congestive heart failure, arrhythmias, myocardial infarctions, and congenital heart disease, acquired or inherited thrombophilic condition, severe blood hypertension (≥160/100 mmHg), diabetes mellitus, recent surgery and/or cancer and age ≤ 18 years.

All subjects underwent outpatient examination, including an interview on general health information, socio-demographic characteristics, lifestyle, medical histories and anthropometrics measurements. A venous blood sample was collected after overnight fasting and stored at − 80 °C. Biochemical markers, including total cholesterol (Total-Chol), low-density-lipoproteins-associated cholesterol (LDL-Chol), high-density-lipoproteins-associated cholesterol (HDL-Chol) and total serum triglycerides (Tgl) were measured by standard biochemical methods in the Central Core laboratory of the University of Padua. Serum PFOA levels were measured by liquid chromatography coupled with triple quadrupole mass spectrometry (LC-MS/MS) as previously described [21].

Based on geographical distribution of PFAS pollution, subjects were then grouped depending on their residence since birth. According to the inventory data on PFAS pollution by regional authorities (Veneto Region: D.G.R. 2133/2016, Annex A and subsequent modifications. (2016)) a highly exposed and a low exposure area were identified according to the most recent official health surveillance data, dating back to 2018 [Pfas - Sostanze Perfluroalchiliche - Azienda ULSS 8 Berica]. Among the 97 subjects finally included in the study, 59 were resident in highly exposed area (exposed group) whilst 38 lived in the low exposed area (control group).

### 2.3 Liquid chromatography-mass spectrometry

PFOA levels were measured through reversed-phase (RP) liquid chromatography coupled with triple quadrupole mass spectrometry (LC-MS/MS) Agilent Varian 320 (Agilent Technologies, Santa Clara, CA, USA), equipped with an Agilent XDB C-18 column, as previously described [21]. Briefly, patients lyophilized sera or were added with the isotope-labeled internal standards (MPFOA) at fixed concentrations of 5 ppb (MPFOA, Wellington Laboratories, Ontario, Canada). After extraction with 400 μl of methanol, this solution was analysed by LC-MS/MS (Electro Spray Ion Source with negative ion mode. Needle voltage 5000 V and drying gas temperature 300 °C, with drying gas pressure was 22 psi and nebuliser pressure was 55 psi. Capillary voltage 40 and CID gas 1,5 mbar). The Limit of Quantification was 0.2 ng/mL.

### 2.4 Cell cultures

Human hepatocellular carcinoma cell line HepG2 was a kind gift from Dr Santina Quarta (Department of Medicine, University of Padova, Padova, Italy). Cells were maintained in minimum essential medium (MEM), supplemented with 10% fetal bovine serum (FBS), antibiotics penicillin-G/streptomycin (Gibco-Thermo Fisher) and antifungal amphotericin B (Euroclone, Milano, Italy). Cells were propagated when 90% confluence was reached, and medium was refreshed every other day.

In stimulation experiments, cells were starved overnight in MEM supplemented with 10% charcoal-treated FBS (Thermo Scientific) and then exposed for 24 h to either PFOA, PFOS or C6O4, singularly at a concentration ranging from 0 ng/mL to 100 ng/mL, diluted in starving medium. For LDL-uptake assay, immediately after the exposure to PFAS, cells were washed with phosphate buffer saline (PBS) and then incubated for 2 h with Bodipy-dye-labeled LDL. Subsequently, cells were washed with PBS, fresh medium was added and samples fluorescence at 515 nm was quantified by Victor X3 Fluorimeter (Perkin Elmer, Waltham, MA, USA). The possible involvement of PCSK9 on LDL uptake upon the exposure to PFAS was assessed by exposing cells for 24 h to PFOA, PFOS or C6O4, each one at the concentration of 100 ng/mL, in absence or presence of human recombinant PCSK9 at the concentration of 5 μg/mL. The protein expression of LDL-R was then evaluated by western blot analysis. The possible impact of PFAS exposure on the equilibrium of LDL-R trafficking, between the exposed portion on the membrane and the cytoplasmic pool, was evaluated by a subcellular protein fractionation approach at basal condition, after the exposure to PFOA, PFOS or C6O4, each one at the concentration of 100 ng/mL, and upon stimulation with sphingosine, a recognized inducer of endocytosis, at the final concentration of 10 μM an added during the latest two hours of exposure [22], [23], [24].

Data, obtained in triplicate per experimental condition, were normalized on control conditions in which PFAS were omitted.

### 2.5 Western blot

Cells exposed for 24 h to PFOA, PFOS and C6O4 as described above, were successively washed in cold PBS and then harvested by scraping. Whole cell pellets were then resuspended in RIPA lysis buffer (NaCl 150 mM, Nonidet P-40 1%, Sodium Deoxycholate 0.5%, Sodium dodecyl-sulphate 0.1%, TRIS 25 mM, pH 7.4), containing protease inhibitor cocktail at the concentration suggested by the manufacturer, and then sonicated for 20 s at 4 °C. Subcellular fractionation was carried out using a commercially available protein fractionation kit, following the manufacturer's instructions for sequential isolation of cellular compartments from an average of 5 × 10^6^ cells [24]. The kit included specific cytoplasmic and membrane protein extraction buffers, which were used to investigate protein localization and redistribution in this study. Samples total protein concentration was estimated using the BCA Protein Assay kit (SERVA). Protein samples were denatured by boiling for 10 min in loading buffer containing sodium dodecyl sulphate and 2-β-mercaptoethanol, and then fractionated using SDS-PAGE gel, blotted onto nitrocellulose membranes and blocked with EveryBlot Blocking Buffer (all from Bio-Rad Laboratories Inc., Milano, Italy). Blots were incubated overnight at 4 °C with the primary antibody (1:500) whose primary immunoreaction was detected by the incubation with a properly diluted HRP-conjugated secondary antibody (1:1000, Cell Signaling Tech.) and visualized with Chemiluminescent Substrate LumiGLO Reserve (Seracare, Milford, MA, USA). Signals were acquired with the Chemidoc XRS System (Bio-Rad). β-Actin (Sigma-Aldrich, cat # 127M4866V) and GAPDH (GeneTex, cat # GTX100118) served as internal control.

### 2.6 Statistical analyses

Statistical analysis of data was conducted with SPSS 21.0 for Windows (SPSS, Chicago, IL, USA). The comparison between two groups of data was performed by Student's t-test after acceptance of normal distribution of the data with the Kolmogorov–Smirnov test. One-way ANOVA with Bonferroni correction was used for the comparison of more than 2 groups of data. Values of P < 0.05 were considered as statistically significant at two-tailed tests.

## 3 Results

### 3.1 Environmental exposure to PFAS associates with altered lipidemic profile

**Fig. 1:**
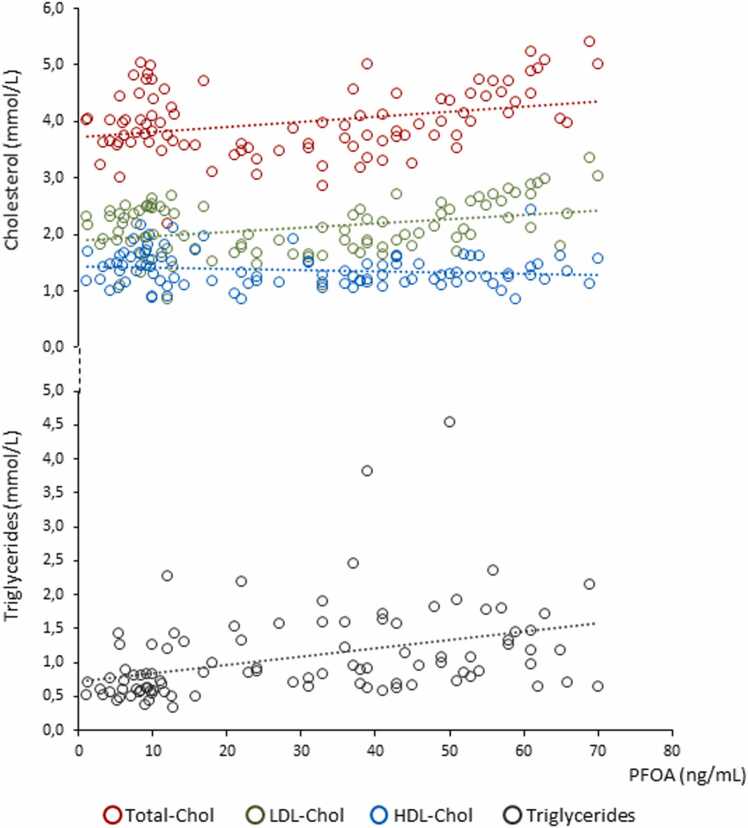
Correlation analysis between serum levels of perfluoro-octanoic acid (PFOA) and total cholesterol (Total-Chol), low-density-lipoproteins-associated cholesterol (LDL-Chol), high-density-lipoproteins-associated cholesterol (HDL-Chol) and total serum triglycerides (Tgl) in 97 study participants. Data regression curves for each correlation are reported as dotted lines.

Demographic and clinical data of the 97 study participants, distinguished according to the low (CTRL, N = 38) and high (Exposed; N = 59) environmental exposure to perfluoroalkyl substances, are reported in Table 1. Compared to CTRL subjects, exposed participants showed no difference in terms of age, BMI, total serum cholesterol (Total-Chol) and LDL-Chol. On the other hand, exposed participants showed lower levels of serum HDL-Chol and higher serum concentration of Tgl together with, as expected, higher serum levels of PFOA. Importantly, serum levels of PFOA were positively and significantly correlated with Total-Chol (*ρ* = 0.312, P = 0.002), LDL-Chol (*ρ* = 0.333, P = 0.001) and Tgl (*ρ* = 0.375, P < 0.001; Fig. 1). These correlations were maintained also upon the correction for BMI values (respectively: Total-Chol *ρ* = 0.315, P = 0.002; LDL-Chol *ρ* = 0.346, P = 0.001; Tgl *ρ* = 0.381, P < 0.001).

**Table 1:** Demographic data and serum lipid levels of the 97 study participants, distingushed according to the residence with low (CTRL) and high (Exposed) environmental exposure to perfluoro-alkyl substances.

	CTRL (N = 38)	Exposed (N = 59)	P value
Age (years)	20.1 ± 1.3	19.8 ± 1.4	0.292
BMI (kg/m^2^)	24,39 ± 4,56	25,39 ± 4,74	0.308
Total-Chol (mmol/L)	4.07 ± 0.51	4.0 ± 0.64	0.583
LDL-Chol (mmol/L)	2.15 ± 0.39	2.13 ± 0.49	0.859
HDL-Chol (mmol/L)	1.5 ± 0.33	1.31 ± 0.26	**0.002**
Triglycerides (mmol/L)	1.63 ± 0.64	3.04 ± 1.7	**< 0.001**
PFOA (ng/mL)	8.43 ± 3,69	43,25 ± 14,63	**< 0.001**
Total-Chol ≥ 5.2 mmol/L [N, (%)]	0 (0%)	2 (3.4%)	0.518^a^
LDL-Chol ≥ 3.3 mmol/L [N, (%)]	2 (5.3%)	14 (23.7%)	**0.023**^a^
HDL ≤ 1.03 mmol/L [N, (%)]	3 (7.9%)	5 (8.5%)	0.919^a^
Triglycerides ≥ 1.7 mmol/L [N, (%)]	0 (0%)	11 (18.6%)	**0.006**^a^

Abbreviations: body mass index, BMI; cholesterol, Chol; low-density lipoproteins, LDL; high-density lipoproteins, HDL; perfluoro-octanoic acid, PFOA.

a P value for chi-square test

Subsequently, the subjects were further divided in two groups depending on their serum lipid levels being below or beyond the cardiovascular diseases safety threshold.The latter, defined “at risk”, having respectively Total-Chol ≥ 5.2 mmol/L, LDL-Chol ≥ 3.3 mmol/L, HDL ≤ 1.03 mmol/L and Tgl ≥ 1.7 mmol/L. Compared to CTRL subjects, the prevalence of exposed participants at risk was significantly higher for LDL-Chol (5.3% CTRL *vs* 23.7% exposed, P = 0.023) and Tgl (0% CTRL *vs* 18.6% exposed, P = 0.006).

### 3.2 In vitro effect of PFAS exposure on LDL-uptake and involvement of PCSK9-related function

**Fig. 2:**
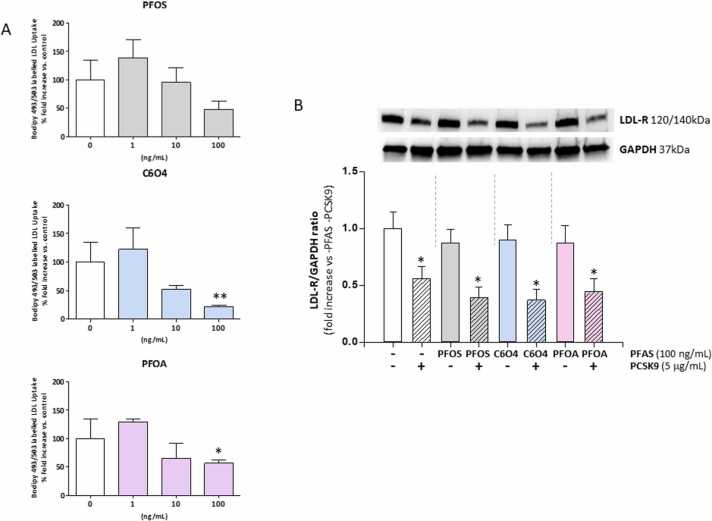
A) Analysis of Bodipy-dye-labelled low-density-lipoproteins (LDL) uptake by HepG2 cell line upon 24 h exposure to perfluoro-octane sulfonic acid (PFOS), acetic acid, 2,2-difluoro-2-((2,2,4,5-tetrafluoro-5(trifluoromethoxy)− 1,3-dioxolan-4-yl)oxy)-ammonium salt (1:1) (C6O4) or acid perfluoro-octanoic acid (PFOA), at the indicated concentrations. Results are the mean value of a triplicate, reported as the percentage fold increase compared to the control (0 ng/mL). Significance: * =P < 0.05 and * *=P < 0.01 *vs* corresponding control. B) Western blot analysis of LDL-receptor (LDL-R) expression in HepG2 cells upon 24 h exposure to PFOS, C6O4 or PFOA, each at the concentration of 100 ng/mL, in presence or absence of human recombinant Proprotein Convertase Subtilisin/Kexin 9 (PCSK9) at the concentration of 5 μg/mL. Glyceraldehyde-3-phosphate dehydrogenase (GAPDH) was used as reference. In control conditions, both perfluoro-alkyl acids (PFAAs) and PCSK9 were omitted. Representative images of three independent experiments are reported. LDL-R to GAPDH band density was normalized on control conditions. Significance: * =P < 0.05 *vs* control condition.

The possible effect of the short-term exposure to PFAS on LDL-uptake in hepatic cells was investigated *in vitro*. Starved HepG2 cells were exposed for 24 h to PFOA, PFOS or C6O4, at concentrations ranging from 0 ng/mL (CTRL) to 100 ng/mL an the extent of Bodipy-dye-labeled LDL uptake was then assessed (Fig. 2A). Compared to CTRL condition, exposure to PFOA or C6O4 at a concentration of 100 ng/mL was associated with a significantly lower LDL uptake signal (respectively: CTRL 100.0 ± 35.1%; PFOA 56,9 ± 4,9%, P = 0.023 *vs* CTRL and C6O4 22,1 ± 1,7%, P = 0.0085 *vs* CTRL). Exposure to lower concentrations of the two compounds showed no significant effects compared to CTRL. On the other hand, exposure to PFOS was associated with a trend towards the reduction of LDL-uptake, but no significant effect was observed (all p-values >0.05).

Subsequently, the possible involvement of PCSK9 on the observed reduction of LDL uptake upon the exposure to PFAS, was evaluated (Fig. 2B). The exposure to any PFAS was not associated with a variation of LDL-R protein expression, compared to the not exposed control. On the other hand, LDL-R expression levels were almost halved upon incubation with PCSK9 alone. Interestingly, exposure to PFOS, C6O4 or PFOS showed no further variation of the PCSK9-dependent degradation of LDL-R, ruling out any major involvement of this pathway on the altered LDL-uptake observed during the exposure to PFAS.

### 3.3 Exposure to PFAS impairs the endocytosis-mediated cell trafficking of LDL-R

**Fig. 3:**
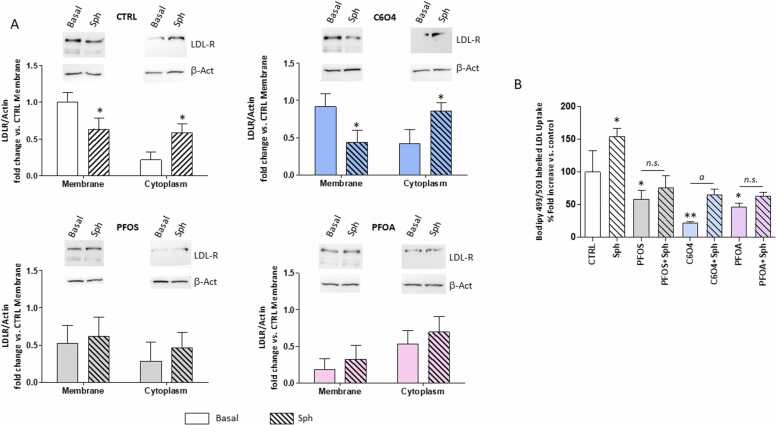
A) Western blot analysis of the low-density-lipoproteins-receptor (LDL-R) partitioning between the membrane and cytoplasmic subcellular fractions of HepG2 cells, isolated as detailed in the method section. Cells were exposed for 24 h to perfluoro-octane sulfonic acid (PFOS), acetic acid, 2,2-difluoro-2-((2,2,4,5-tetrafluoro-5(trifluoromethoxy)− 1,3-dioxolan-4-yl)oxy)-ammonium salt (1:1) (C6O4) or acid perfluoro-octanoic acid (PFOA), without (Basal) or upon the treatment for 2 h with sphingosine (Sph). In control condition (CTRL), expsosure to perfluoro-alkyl acids (PFAAs) was omitted. β-Actin (β-Act) was used as reference. Representative images of three independent experiments are reported. LDL-R to β-Act band density was normalized on basal CTRL. Significance: * =P < 0.05 *vs* corresponding basal. B) Analysis of Bodipy-dye-labelled LDL uptake by HepG2 cell line upon 24 h exposure to PFOS, C6O4 or PFOA, without (Basal) or upon the treatment for 2 h with Sph. In control condition (CTRL) any exposure or treatment was omitted.Results are the mean value of a triplicate, reported as the percentage fold increase compared to the CTRL. Significance: * =P < 0.05 and * *=P < 0.01 *vs* CTRL; *a*=P < 0.05 among indicated conditions; *n.s.* = not significant.

The uptake of LDL from the extracellular environment is strictly linked to the amount of LDL-R exposed on cell surface and, in turn, to the extent of internalized LDL/LDL-R complex internalized by endocytosis, to the trafficking of the complex to degradation in lysosomes, and to the recycling of the receptor back to the cell surface [25]. The possible impact of PFAS exposure on the equilibrium of LDL-R trafficking, was the assessed (Fig. 3A). As expected, in not exposed control conditions, the treatment with sphingosine was associated with a significant decrease of LDL-R levels in cell membranes and a corresponding increase of protein signal in the cytoplasm fraction (respectively: P = 0.0322 basal *vs* sphingosine membrane and P = 0.0174 basal *vs* sphingosine cytoplasm). A similar response to sphingosine was observed upon the exposure to C6O4 (respectively: P = 0.0236 basal *vs* sphingosine membrane and P = 0.0255 basal *vs* sphingosine cytoplasm). Differently, the exposure to either two legacy PFAS representatives, PFAS an PFOA, was associated with a non-significant translocation of LDL-R signal from membrane to cytoplasm upon treatment with sphingosine (respectively: PFOS P = 0.676 basal *vs* sphingosine membrane and P = 0.4.68 basal *vs* sphingosine cytoplasm; PFOA P = 0.3742 basal *vs* sphingosine membrane and P = 0.3627 basal *vs* sphingosine cytoplasm).

To investigate the functional implications of this finding, we evaluated the impact of sphingosine treatment on the cell uptake of Bodipy-dye-labeled LDL in not exposed control condition and upon exposure to PFOS, C6O4 or PFOA, at the concentration of 100 ng/mL,for 24 h (Fig. 3B). In CTRL conditions, sphingosine treatment was associated with a significant increase of LDL uptake compared to basal levels (respectively 100,00 ± 32,69 basal *vs* 154,37 ± 12,91 sphingosine, P = 0.0110). Despite the exposure to C6O4 was associated with a reduction of LDL uptake at basal conditions, sphingosine treatment induced a significant retrieval of the LDL uptake function (respectively 22,10 ± 1,73 basal *vs* 65,33 ± 8,31 sphingosine, P = 0.0468). However, neither cells exposed to PFOS nor those exposed to PFOA showed a response to sphingosine in terms of increased LDL uptake, when compared to basal conditions (respectively PFOS 58,24 ± 14,01 basal *vs* 75,90 ± 19,00 sphingosine, P = 0.8532; PFOA 46,98 ± 4.98 basal *vs* 62,90 ± 5.8 sphingosine, P = 0.903).

## 4 Discussion

In this study, we provide evidence of a possible novel mechanism of lipid metabolism disruption, involving the unbalance of LDL-R cell trafficking with the apparent independence from the PCSK9-mediated pathway.

There is a considerable amount of evidence in regard of the association between serum cholesterol levels and exposure to legacy PFAS in humans. Avery recent meta-analysis from Liu et al., evaluating 29 publications, showed that every interquartile-range increase of PFOA was associated with a 2.1 mg/dL increase in total Chol, 1.3 mg/dL increase in Tgl, and a 1.4 mg/dL increase in LDL-Chol [26]. Similar, though less obvious, associations have been documented for PFOS [26]. More precisely, epidemiologic association data of serum Chol to PFOA show a steep correlation up to PFOA serum levels of 10 ng/mL, a milder though significant association up-to 100 ng/mL and a nearly saturated trend thereafter [5], [27]. In the present study, we also show a significant association of serum levels of PFOA to both cholesterol and Tgl levels, with no apparent role of BMI as confounding factor. It should be noted also the peculiarity of the subjects recruited in the study, being all slightly above the age of majority and distinguished by residence in areas with different environmental exposure to PFOA. In this regard, a significantly increased proportion of exposed subjects had cholesterol levels close to the cardiovascular risk threshold. Although it was not possible to conduct a screening for genetic factors of familial hypercholesterolemia, this evidence supports an independent role of PFOA exposure as metabolic risk factors and, consequently, as a cardiovascular risk factor even in the younger age groups [21], [28], [29], [30].

While the available evidence in animal models is consistent with human data, the underlying mechanism driving the aforementioned causal relationship is currently under debate [5]. Classical mechanisms of endocrine disruption have been claimed in studies aimed to address the possible involvement of peroxisome proliferator-activated receptor α (PPARα) in the altered lipid metabolism associated with the exposure to legacy PFAS. In 2017, Das et al. showed that an increased lipid accumulation in mice liver was observed upon the exposure either PFOA, perfluorononanoic acid (PFNA), or perfluorohexane sulfonate (PFHxS), but in PPARα-null animal, liver steatosis was a feature of PFNA and PFHxS exposure, highlighting also major structure-related toxicology outcomes [31]. On the other hand, through a transcriptomic approach, Rosen et al. in 2017 highlighted the involvement of other nuclear receptors in addition to PPARα, such as constitutive activated receptor (CAR), estrogen receptor α (ERα), and PPARγ [31]. Regarding cholesterol metabolism, PFOA showed to be ineffective in inducing hyper-cholesterolemia in PPARα-null mice suggesting the involvement of different pathways for the two classes of lipids [32], [33]. It should be also noted that protein PPARα levels in human livers are lower than those in rodents and PFAS are acknowledged as weaker agonists of human PPARα than of the rodent’s receptor [31], [33], [34], [35].

On these premises, other mechanistic models should be invoked to explain the disrupting role of PFAS on lipid metabolism. In this frame, we recently focused on an upstream disrupting effect model of these chemicals, associated to their surfactant properties, and taking place at the plasma membrane level [36]. Accordingly, we recently showed that PFOA was able to perturb the insulin-receptor signaling by altering the balance of complex formation with membrane glycolipids associated with upstream receptor uncoupling [37]. Membranes glycolipids, such as gangliosides, are also mediators of cell endocytosis, a key process involved in multiple functions of cellular physiology, including the LDL uptake *via* LDL-receptor [38]. We found that short term exposure to PFAS associates to altered LDL uptake in hepatocyte cell model and that this early effect is related to the possible unbalance of the LDL-receptor trafficking as evidenced by subcellular fractionation experiments. In addition, sphingosine, a known triggering agent of endocytosis [22], [23], was shown to increase basal LDL-uptake but was essentially ineffective at restoring this cellular function in cells exposed to legacy PFAS such as PFOS and PFOA. Most importantly, the PFAS exposure showed no differential pattern of PCSK9-dependent degradation of LDL-R, ruling out a major role of this pathway. Interestingly, the new generation perfluoroalkyl substance C6O4 showed to impair basal LDL-uptake but this effect was significantly offset by sphingosine. At the basis of this phenomenon there could be a differential interaction with membrane lipids by C6O4, compared to legacy PFAS: more superficial for the former and intercalating for the latter, supporting a structure-activity effect already highlighted in previous studies [31]. In support of this possible mechanistic hypothesis, Frawley et al. recently investigated in a murine model the immunotoxic and hepatotoxic effects of another linear legacy-PFAS: the perfluoro-n-decanoic acid (PnDA) [39]. Aside of a prevalent hepatomegalia, authors documented that exposure to PnDA was associated with splenic atrophy and impaired humoral- and cell-mediated immunity in spite of no changes in leukocyte subpopulations. Since phagocytosis was decreased in liver tissue macrophages, authors suggested a major interference of PnDA on endocytosis-related processes in resident immune cells of the liver [39].

We acknowledge some drawbacks in the present study, such as the absence of a genetic characterization of the study cohort and the absolutely preliminary observation of the effects of PFAS on the cellular redistribution of LDLR. In addition, we did not quantify PFOA and C6O4 in subject’s sera. In this regard, it should be noted that previous data from our group outlined a minor contribution of PFOS to total PFAS levels in the geographical area under analysis [40]. On the other hand, although some preliminary data on environmental detection of C6O4 are getting available, there are as yet no clear data about the serum detection of the compound in the general population [41]. On these bases we decided to focus on PFOA as the major PFAS pollutant in the Veneto Region. Further studies will be needed to understand the exact molecular target of these compounds in the complex mechanism regulating endocytosis.

In conclusion, we confirmed that environmental exposure to PFAS, and particularly the legacy compounds PFOA and PFOS, associates with altered serum levels of LDL-cholesterol and triglycerides. Furthermore, exposure of hepato-carcinoma cell line to PFOA and PFOS was associated with an altered pattern of LDL-R partition between membrane and cytoplasm which was not reversed by sphingosine as an endocytosis inducer. These data suggest a novel mechanism of altered lipid trafficking associated with the exposure to legacy PFAS.

## Funding

The study was funded by a grant from Solvay Specialty Polymers Italy S.p.A. The funder had no role in the design and conduct of the study; collection, management, analysis, and interpretation of the data, or approval of the manuscript; and decision to submit the manuscript for publication.

## CRediT authorship contribution statement

**Iva Sabovic:** Investigation. **Maria Giovanna Lupo:** Investigation. **Ilaria Rossi:** Investigation. **Stefano Dall’Acqua:** Investigation. **Nicola Ferri:** Writing – review & editing. **Andrea Di Nisio:** Visualization, review & editing. **Alberto Ferlin:** Writing – review & editing, Project administration. **Carlo Foresta:** Conceptualization, Funding acquisition. **Luca De Toni:** Conceptualizations, Visualization, Writing.

## Declaration of Competing Interest

The authors declare that they have no known competing financial interests or personal relationships that could have appeared to influence the work reported in this paper.

## Data Availability

Data will be made available on request.
